# Heterogeneity of Size and Toxin Distribution in *Aggregatibacter actinomycetemcomitans* Outer Membrane Vesicles

**DOI:** 10.3390/toxins16030138

**Published:** 2024-03-07

**Authors:** Justin B Nice, Shannon M. Collins, Samuel M. J. Agro, Anxhela Sinani, Spencer D. Moros, Leah M. Pasch, Angela C. Brown

**Affiliations:** 1 Department of Chemical and Biomolecular Engineering, Lehigh University, Bethlehem, PA 18015, USA; 2 Department of Bioengineering, Lehigh University, Bethlehem, PA 18015, USA

**Keywords:** *Aggregatibacter actinomycetemcomitans*, outer membrane vesicles (OMVs), leukotoxin, heterogeneity

## Abstract

*Aggregatibacter actinomycetemcomitans* is a Gram-negative bacterium associated with localized aggressive periodontitis as well as some systemic diseases. The strains of *A. actinomycetemcomitans* most closely associated with disease produce more of a secreted leukotoxin (LtxA) than isolates from healthy carriers, suggesting a key role for this toxin in disease progression. LtxA is released into the bacterial cytosol in a free form as well as in association with the surface of outer membrane vesicles (OMVs). We previously observed that the highly leukotoxic *A. actinomycetemcomitans* strain JP2 produces two populations of OMVs: a highly abundant population of small (<100 nm) OMVs and a less abundant population of large (>300 nm) OMVs. Here, we have developed a protocol to isolate the OMVs produced during each specific phase of growth and used this to demonstrate that small OMVs are produced throughout growth and lack LtxA, while large OMVs are produced only during the exponential phase and are enriched with LtxA. Our results indicate that surface-associated DNA drives the selective sorting of LtxA into large OMVs. This study provides valuable insights into the observed heterogeneity of *A. actinomycetemcomitans* vesicles and emphasizes the importance of understanding these variations in the context of bacterial pathogenesis.

## 1. Introduction

The Gram-negative bacterium *Aggregatibacter actinomycetemcomitans* is associated with aggressive forms of periodontitis, particularly in adolescents [1,2,3,4], as well as systemic infections such as endocarditis [5]. This organism produces several virulence factors [6], including a leukotoxin (LtxA), which selectively kills white blood cells [7], thus inhibiting an effective host immune response. The amount of LtxA that is produced by different strains of *A. actinomycetemcomitans* has been correlated to the strains’ association with periodontal disease [2,4,8,9,10,11], suggesting an important function of this virulence factor in pathogenesis, especially in juveniles.

LtxA is released from the bacterium in both a “free” form as well as in association with outer membrane vesicles (OMVs) [12,13]. Like other members of the repeats-in-toxin (RTX) family of proteins [14], LtxA is secreted via a type 1 secretion system (T1SS) across both the inner and outer membranes of the bacterium in a single step [15,16]. Once secreted, free LtxA interacts with host cell membranes through the recognition of both cholesterol [17,18] and an integrin receptor, lymphocyte function-associated antigen-1 (LFA-1) [19,20,21]. In contrast, OMV-associated LtxA is delivered to host cells in an LFA-1- and cholesterol-independent manner [12]. We and others have found that LtxA is located on the surface of OMVs [12,22], consistent with prior reports that LtxA has an affinity for the bacterial cell surface, reported to be driven by interactions with lipopolysaccharide (LPS) [23] and surface-associated DNA [22,24], in a manner that is sensitive to environmental conditions, such as pH [15] and iron concentration (FeCl_3_) [25]. Thus, it appears that after secretion via the T1SS, some of the toxin associates with the bacterial cell surface, localizing it to the OMV surface following vesicle formation.

Importantly, the OMVs released by *A. actinomycetemcomitans* are enriched in LtxA relative to the concentration on the outer membrane (OM) [12,26], suggesting some type of sorting mechanism to promote OMV association. The factors regulating this enrichment remain unclear; however, it could be related to the reported variation in lipid composition between *A. actinomycetemcomitans* OMVs and the OM [26]. This variation is consistent with other reports in the literature showing that although OMVs are derived from the bacterial OM, significant differences in protein and lipid composition between the OMV and OM can exist, indicating that certain molecules localize to the OMV, while others remain on the OM [27,28,29,30,31].

In addition to compositional differences between the OM and OMVs, several groups have reported that OMVs released by a single organism can exhibit heterogeneous physical properties, particularly size [32,33,34,35]. Interestingly, *Helicobacter pylori* was observed to produce a heterogeneous population of OMVs, with diameters ranging from 20 to 450 nm. The separation of these OMVs by density gradient centrifugation (DGC) demonstrated that smaller OMVs had a less diverse protein content than larger OMVs, and the two populations of OMVs entered host cells via different mechanisms [32]. In our previous work, we noted that the JP2 strain of *A. actinomycetemcomitans* likewise releases heterogeneous OMVs with a bimodal size distribution consisting of a minority population with diameters of approximately 325 nm and a majority population with diameters of approximately 100 nm [12].

In this investigation, our focus was on delving deeper into the heterogeneity of *A. actinomycetemcomitans* OMVs, particularly in relation to the growth phase. Previous studies have faced limitations in collecting OMVs from each growth phase, primarily due to the challenges involved. Typically, OMVs are isolated during the late exponential phase to maximize yield, but this approach results in preparations containing OMVs from all growth phases. To overcome this hurdle, we developed a protocol to efficiently separate OMVs produced during specific growth phases. This innovative method allowed us to explore the distinct properties of OMVs generated at each growth phase. Utilizing this protocol, we found that *A. actinomycetemcomitans* releases small OMVs consistently throughout growth, while large OMVs are specifically released during the late exponential phase. Importantly, OMVs produced in the late exponential phase are enriched in LtxA and demonstrate activity against host immune cells. Finally, we observed that the association of LtxA with *A. actinomycetemcomitans* OMVs is mediated by interactions with surface-associated DNA.

## 2. Results

### 2.1. A. actinomycetemcomitans OMV Toxicity and Size Vary with Growth Phase

Previously, we reported that the highly leukotoxic strain of *A. actinomycetemcomitans* JP2 releases LtxA in both a free form as well as in association with OMVs; in both forms, the toxin is active against host immune cells [12]. In that work, OMVs were collected from the late exponential phase, following standard OMV purification protocols. Because OMV production is often low, it is common to purify OMVs from the late exponential phase to maximize the resulting yield of vesicles. However, recent work has observed significant variations in OMVs’ physical properties and composition throughout growth [33,36,37], and we therefore sought to investigate whether the toxin composition of OMVs changes as the bacteria grow.

**Figure 1:**
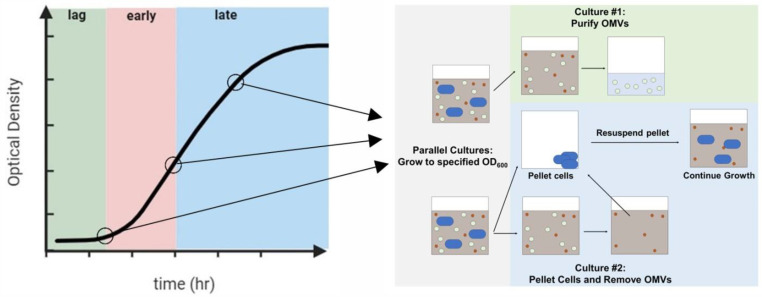
Phases of bacterial growth from which OMVs were purified. Two bacterial cultures were grown in parallel to a specified OD_600_. At that point, the OMVs were collected from the first culture. The second culture was pelleted, and the OMVs were removed; the OMV-free supernatant was used to resuspend the pelleted cells. This process was continued through the late exponential phase.

In previously reported studies aimed at investigating the changes in OMV size with growth phase, no attempts were made to remove OMVs from earlier phases; as a result, OMVs collected from the late exponential phase represented those produced during all growth phases up to and including that point. To better isolate variations throughout growth, we developed an approach to remove OMVs from the culture at specific time points without disrupting bacterial growth using a parallel culture strategy (Figure 1). The culture was grown to the specified time point, at which point, the cells were pelleted, and the vesicles produced up to that point were removed from the supernatant. The resulting OMV-free supernatant was then used to resuspend a bacterial pellet that had been grown in parallel, and this new culture was grown until the next time point was reached. The process was continued through the late exponential phase. We verified that the growth of the bacterial cells was not significantly affected by the removal of the OMVs (Figure S1).

**Figure 2:**
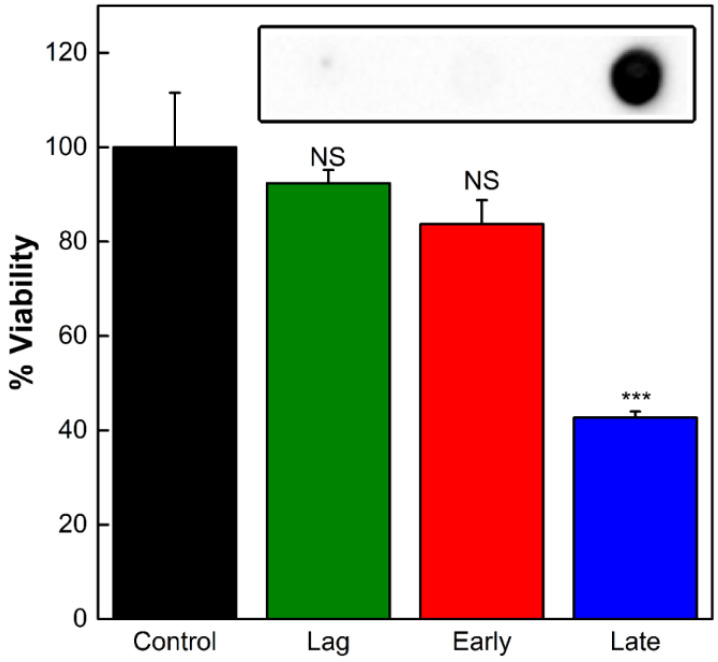
Activity of OMVs produced during different stages of growth. The viability of THP-1 cells was assessed after 16 h of incubation with OMVs collected from the lag, early exponential, or late exponential growth phases. Each data point represents the mean (n = 3) ± standard deviation. NS, not significant; ***, *p* < 0.001 relative to untreated (“control”) cells. Inset: an immunoblot for LtxA in OMVs collected from each of the three phases.

To investigate the variations in LtxA composition between the OMV populations, we first studied the effect of OMVs produced during the lag phase, early exponential, and late exponential phases on THP-1 cell viability. Neither the OMVs from the lag phase nor those from the early exponential phase caused any decrease in THP-1 cell viability relative to untreated cells. In contrast, the OMVs purified from the late exponential phase mediated a decrease in cell viability of approximately 55% (Figure 2). We then measured the concentrations of LtxA associated with the OMVs produced during each of these different growth phases using an immunoblot assay with an anti-LtxA antibody [38]. As shown in the inset of Figure 2, the OMVs produced during the lag and early exponential phases contained nondetectable amounts of LtxA, while the OMVs produced during the late exponential phase contained a high concentration of LtxA. This observation that OMVs produced later in growth contain more LtxA and are more toxic to host cells is consistent with a previous report that LtxA production peaks by the end of the exponential phase, decreasing when the bacteria enter the stationary phase [15]. We also observed that LtxA production is initiated during the exponential phase of growth (Figure S2).

Because of this variation in toxin composition in the various growth phases and our past demonstration that LtxA is more abundant on large OMVs than on small OMVs, we hypothesized that small and large OMVs might be produced at different times during growth. We therefore next investigated variations in *A. actinomycetemcomitans* OMV size during different growth phases to detect any correlations with LtxA composition.

**Figure 3:**
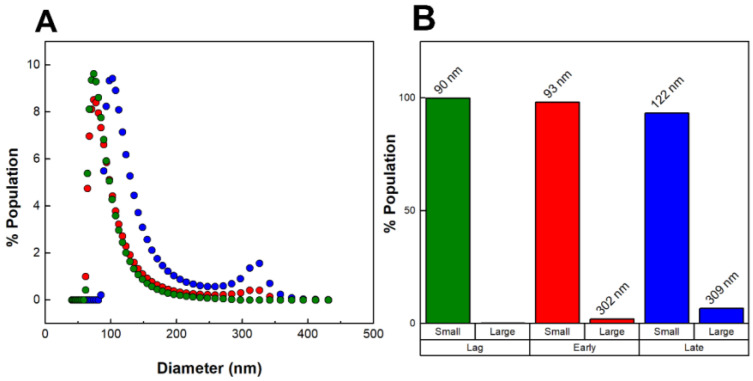
Size heterogeneity of OMVs produced throughout bacterial growth. (**A**) DLS number-weighted distribution of diameters of OMVs collected from the lag phase (green), early exponential phase (red), and late exponential phase (blue). (**B**) Distribution of “small” (<250 nm) and “large” (>250 nm) OMVs. The average diameters are noted above each population group. The data shown are representative results from n = 3 trials.

Figure 3A shows the DLS number-weighted distributions of OMV diameters for the three OMV preparations (lag phase, early exponential phase, and late exponential phase). OMVs produced during the lag phase were found to be small (90 nm in diameter) and homogeneous; no large OMVs were detected. OMVs produced during the early exponential phase contained a very small fraction of large OMVs (300 nm) in addition to the 90 nm population, while OMVs produced during the late exponential phase consisted of an even greater fraction of large OMVs. As we observed previously, the populations of OMVs were distinct, with one smaller population below 250 nm in diameter and one large population with diameters larger than 250 nm. The distributions of small and large OMVs in each of the three preparations are shown in Figure 3B. The percentage of large OMVs increased with growth time, with the OMVs produced during the late exponential phase consisting of 7% large OMVs.

It is important to stress that our “late exponential” OMVs represent only those OMVs produced during the exponential phase, as all OMVs produced during the lag and early exponential phases were removed from the culture at the beginning of the exponential phase. This approach enables us to distinguish between OMVs produced early in growth and those produced during later stages of growth. Our results thus demonstrate that the large OMVs we have observed previously are produced only during the exponential phase of growth, while the smaller OMVs are produced throughout growth.

### 2.2. LtxA Does Not Mediate Large OMV Formation

Our observations that OMVs produced during the late exponential phase contain more LtxA and consist of a greater fraction of large OMVs are consistent with our prior finding that LtxA is more abundant on large OMVs [39,40,41]. Knowing that LtxA is located on the surface of OMVs [12] and that it is a membrane-active protein that is able to induce curvature in model membrane systems [42], we hypothesized that the preferential association of LtxA with large OMVs might be mediated by the production of LtxA during the exponential phase. In other words, as LtxA is secreted, some may associate with the bacterial surface, inducing a membrane curvature that initiates the formation of large OMVs. To test this hypothesis, we compared the sizes of OMVs produced by JP2 (highly leukotoxic, serotype b [43]) with those produced by AA1704, a non-LtxA-expressing isogenic mutant of JP2 [44] (also serotype b), as well as *A. actinomycetemcomitans* strain 33384, a minimally leukotoxic, serotype c strain [45]. The serotypes of *A. actinomycetemcomitans* are defined by the structure of the O-polysaccharide component of their LPS [46]. Thus, this combination of strains allowed us to compare the effects of LtxA and the structure of LPS on large OMV formation.

**Figure 4:**
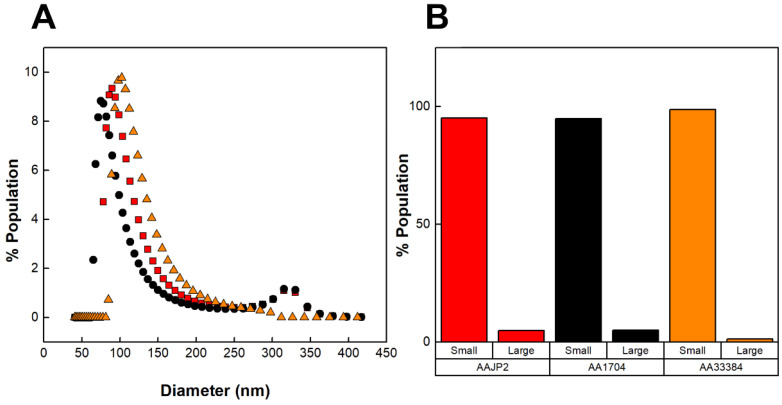
DLS distributions of OMVs produced by various strains of *A. actinomycetemcomitans*. (**A**) Number-weighted DLS distributions of OMVs. OMVs produced by JP2 (black circles), AA1704 (red squares), and ATCC 33384 (orange triangles). (**B**) Concentrations of large (>250 nm) and small (<250 nm) OMV subpopulations, calculated from the DLS distributions. The data shown are representative results from n = 3 trials.

DLS was used to determine the distribution of the diameters of the vesicles produced by the three strains of *A. actinomycetemcomitans* (Figure 4A). Figure 4B represents the concentrations of small (<250 nm) and large (>250 nm) OMVs calculated from the DLS distributions. We found that although AA1704 does not produce LtxA, it was able to produce large OMVs. In contrast, ATCC 33384 (serotype c) did not produce any large vesicles. These results demonstrate that, contrary to our hypothesis, LtxA itself is not directly involved in the production of large OMVs.

### 2.3. LtxA Binding to Large OMVs Is Mediated by Surface-Associated DNA

Previous reports have shown that polymyxin B (PMB) and DNase I treatments removed LtxA from the surface of *A. actinomycetemcomitans* cells and vesicles [22,23,24]. Therefore, we investigated whether the selective sorting of LtxA to large OMVs is mediated by either LPS or surface-associated DNA. We tested both conditions by first pretreating JP2 OMVs collected from the late exponential phase with PMB or DNase I and measuring the resulting changes in the LtxA elution profile by size exclusion chromatography (SEC). We have previously shown that this SEC approach is able to separate large and small *A. actinomycetemcomitans* OMVs [39,41]; thus, we anticipated that a similar approach could be used to detect variations in the LtxA composition of large and small OMVs after PMB or DNase I treatment.

**Figure 5:**
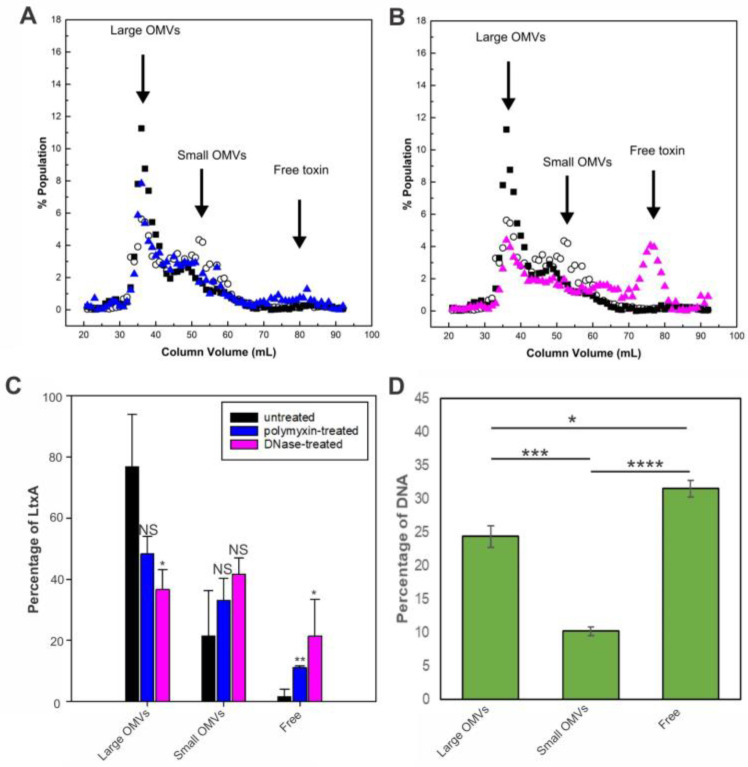
Mechanisms of LtxA binding to OMVs collected from the late exponential phase. (**A**) PMB-treated JP2 OMVs separated by SEC. The LtxA concentration in each fraction after PMB treatment (blue triangles) is shown, along with the LtxA concentration of untreated OMVs (black squares) and the lipid concentration (empty circles). The data shown are representative results from n = 3 trials. (**B**) DNase I-treated JP2 OMVs separated by SEC. The LtxA concentration in each fraction after DNase I treatment (pink triangles) is shown, along with the LtxA concentration of untreated OMVs (black squares) and the lipid concentration (empty circles). (**C**) Percentage of LtxA associated with large OMVs, associated with small OMVs, or unassociated before (black) or after PMB (blue) or DNase (pink) treatment. Each data point represents the mean ± standard deviation. *, *p* < 0.05; **, *p* < 0.01; NS, not significant relative to respective untreated value. (**D**) DNA concentration in fractions containing large OMVs (fractions 30–40), small OMVs (fractions 50–60), and free DNA (fractions 80–90), as measured by TOTO-1 fluorescence. The data shown are representative results from n = 3 trials. Each data point represents the mean ± standard deviation. *, *p* < 0.05; ***, *p* < 0.005; ****, *p* < 0.0001.

Figure 5A shows a representative SEC elution profile after the PMB treatment of the OMVs. The lipid and LtxA concentrations of untreated OMVs are included for reference. Following the PMB treatment, the LtxA concentration in the fractions comprising large OMVs (32–40 mL) decreased slightly, and the LtxA concentration in the fractions corresponding to free LtxA (that is, unassociated with OMVs, 70–83 mL), increased slightly. This result indicates that the PMB-mediated neutralization of LPS removed only a small amount of LtxA from the surface of the large OMVs.

We next investigated the role of surface-associated DNA in the association of LtxA with large OMVs by treating the OMVs with DNase I before separation by SEC. Figure 5B also includes the lipid and LtxA concentrations of untreated OMVs for reference. After the DNase I treatment, the LtxA concentration in the fractions containing large OMVs decreased drastically, while the amount in the fractions containing free LtxA (70–83 mL) increased correspondingly. Figure 5C shows the fraction of LtxA associated with large OMVs, associated with small OMVs, or unassociated (“free”) before and after PMB or DNase treatment for three separate trials. These results demonstrate that LtxA binding to the surface of the large vesicles is mediated by the presence of surface-associated DNA.

The importance of surface-associated DNA was further assessed by measuring the amount of surface-associated DNA in each OMV fraction. Using the membrane-impermeable nucleic acid stain TOTO-1 [47], we found that DNA was concentrated in the fractions containing large OMVs but not in the fractions containing small OMVs (Figure 5D). Additionally, we observed a significant amount of DNA elution in very late fractions after free LtxA, which is likely unassociated DNA that co-purifies with the OMVs.

## 3. Discussion

OMVs have emerged as an important factor in bacterial virulence [26,48,49]. In particular, their role in the delivery of toxins to host cells has been established as an important aspect of virulence. The OMV-mediated transport of toxins enables delivery to the cytoplasm of the host cell [48] in a form that is protected from harsh extracellular conditions, including proteases [26,34,50]. In many cases, it has been observed that the delivery of OMV-associated toxins to host cells differs from that of free toxins [12,13,50]. For example, we have observed that OMV-associated LtxA is delivered to host cells in a cholesterol- and LFA-1-independent manner [12], unlike free LtxA, which requires both of these molecules [17,18,19,20,21]. While the mechanisms by which free toxins are delivered to host cells have been well established, the delivery of OMV-associated toxins to host cells is currently a subject of intense focus. Thus, understanding the nature of the association of toxins with OMVs is essential to elucidating the mechanisms by which these OMVs are produced and interact with host cells.

In prior work, we found that LtxA is located predominately on the larger OMVs produced by the *A. actinomycetemcomitans* strain JP2 [39,40], consistent with a previous study of a different strain of *A. actinomycetemcomitans*, strain D7SS, in which it was found that LtxA was associated with OMVs with reduced density, that is, larger OMVs, compared to another exotoxin, cytolethal distending toxin (Cdt) [35]. The D7SS strain is a nonfimbriated smooth colony mutant of a clinical isolate of *A. actinomycetemcomitans*, suggesting that the heterogeneous sorting of LtxA on OMVs is a common and clinically relevant phenomenon. In addition, the sorting of RTX toxins to certain OMVs may be conserved and related to their secretion mechanism, as it was reported that the RTX toxin α-hemolysin associates preferentially with large *Escherichia coli* vesicles, along with some components of the T1SS machinery [34].

To investigate the process mediating the association of LtxA with larger OMVs, we examined LPS and surface-associated DNA. It has been reported that LtxA can be extracted from JP2 bacterial cells via PMB treatment [23], suggesting that the association of LtxA with the bacterial cell surface is mediated by interactions with LPS. However, we found that only a small amount of LtxA was removed from the OMVs by this treatment. We then investigated the role of OMV surface-associated DNA in this process, as DNA has also been reported to mediate the binding of LtxA to the bacterial cell membrane [22,24]. In this case, we found that the DNase treatment greatly reduced the amount of LtxA associated with the large OMVs, indicating that LtxA binds to DNA on the surface of large OMVs. Consistent with this observation, we found that large OMVs contain much more DNA on their surface than smaller OMVs.

Surface-associated DNA has been reported to be a key feature of OMVs produced by several types of bacteria, including *H. pylori*, *Pseudomonas aeruginosa*, *Porphyromonas gingivalis*, and *Salmonella typhimurium* [51,52]. Bitto et al. observed that this DNA is derived from chromosomal DNA and is released during the exponential phase of growth by actively dividing bacteria [52]. Our observations that OMVs produced during the exponential phase are enriched in LtxA and that LtxA associates with surface-associated DNA are consistent with these findings. Prior work has demonstrated that after secretion via the T1SS, a significant portion of released LtxA remains associated with the *A. actinomycetemcomitans* cell surface. LtxA can be removed by treating the cells with DNase, demonstrating that the affinity of LtxA for the bacterial cell surface is mediated by interactions with surface-associated DNA [22]. Subsequent work showed that this affinity is regulated by electrostatic interactions [24]. Importantly, LtxA has a relatively high isoelectric point (8.2–8.5 [53]), resulting in it being positively charged at neutral pHs and thus able to interact electrostatically with negatively charged DNA. For these reasons, we hypothesized that association with DNA might regulate the localization of LtxA on the surface of outer-membrane-derived OMVs.

Our current work is focused on understanding the biophysical and biochemical processes involved in DNA’s association with large OMVs. We hypothesize that variations in the LPS structures and charge of the two OMV types regulate the binding of DNA. LPS variations between OMVs and the OM have been reported previously for several organisms, including *P. gingivalis* [27], *E. coli* [28], and *P. aeruginosa* [29]. A thin-layer chromatography (TLC) analysis of *A. actinomycetemcomitans* JP2 OMVs revealed the presence of three lipids, which could include LPS, that were not present in the OM of these cells [26]. We suspect that similar variations in the LPS structures of large and small OMVs and the resulting variations in charge might regulate DNA binding to large OMVs. The origin of this DNA is also not currently known; however, Bitto et al. found that DNA associates with OMVs during the exponential phase, and they hypothesized that actively dividing bacteria shed DNA, which then associates with OMVs [52]. We are currently investigating whether this proposed mechanism explains the observed variations in DNA composition in *A. actinomycetemcomitans* JP2 OMVs.

Variations in the properties of OMVs across the growth curve have been documented in numerous bacterial species. Tashiro et al. observed an increase in the size of OMVs produced by *P. aeruginosa* during later stages of growth [36]. Similarly, *H. pylori*-generated OMVs exhibit decreased heterogeneity, with a significant shift in protein composition across different growth phases [33]. Notably, Koning et al. reported the release of two distinct populations of OMVs by *Acinetobacter baumannii*: small OMVs released from the distal ends of bacteria throughout growth and larger (200–500 nm) OMVs released from the septum during the exponential phase [37]. Our findings align with these observations, suggesting that the mechanisms governing the release of OMVs by *A. actinomycetemcomitans* JP2 may involve similar variations.

The diversity in OMV characteristics and the consequential variations in cell uptake imply that different populations of OMVs may serve distinct purposes. Despite the potential significance of such distinctions, limited progress has been made in this area due to the technical challenges associated with conducting comprehensive studies. Notably, difficulties arise from the dynamic changes in OMV properties during bacterial growth, coupled with the relatively low yields of small OMVs during earlier growth phases. To overcome these hurdles, we have developed an innovative approach that facilitates the precise separation of OMVs produced during specific growth phases. By combining this method with our SEC approach to separate subpopulations of OMVs, we have been able to gain a more nuanced understanding of how the physical and chemical properties of *A. actinomycetemcomitans* OMVs evolve throughout growth, allowing us to establish correlations with their functional roles.

## 4. Materials and Methods

### 4.1. Bacterial Culture

The *A. actinomycetemcomitans* strains JP2 (serotype b), ATCC 33384 (serotype c) [45], and AA1704, an isogenic *ltxA* mutant [44] of the JP2 strain, were grown under identical conditions. The growth media consisted of 30 g/L of trypticase soy broth (BD Biosciences, Franklin Lakes, NJ, USA) with 6 g/L of yeast extract (BD Biosciences), 0.4% sodium bicarbonate (VWR, Radnor, PA, USA), 0.8% dextrose (BD Biosciences), 5 µg/mL of vancomycin (Sigma-Aldrich, St. Louis, MO, USA), and 75 µg/mL of bacitracin (Sigma-Aldrich). Cultures were first grown in a candle jar for 16 h at 37 °C, then transferred at a ratio of 3:50 to fresh media and grown for an additional 24 h at 37 °C outside of the candle jar. A 1.5 L culture was grown to the late exponential phase (optical density at 600 nm, OD_600_ of 0.68–0.72).

### 4.2. THP-1 Culture

THP-1 leukocytes (ATCC, Manassas, VA, USA) were maintained at 37 °C in 5% CO_2_ in an RPMI 1640 medium (ThermoFisher Scientific, Waltham, MA, USA), supplemented with 10% fetal bovine serum (FBS, Quality Biological, Gaithersburg, MD, USA) and 0.05 mM of 2-mercaptoethanol (VWR, Radnor, PA, USA).

### 4.3. OMV Purification

To purify OMVs from the late exponential phase, the bacteria were pelleted by centrifugation twice at 10,000× *g* and 4 °C for 10 min. The supernatant was next filtered through a 0.45 µm filter. The bacteria-free supernatant was concentrated using Amicon^®^ 50 kDa filters (MilliporeSigma, Burlington, MA, USA) and then ultracentrifuged at 105,000× *g* and 4 °C for 30 min. Pellets were pooled in phosphate-buffered saline (PBS, pH 7.4), ultracentrifuged again, and resuspended in 1 mL of PBS.

To enable the collection of OMVs from different growth phases, two bacterial cultures were grown in parallel. Lag-phase OMVs were collected after the bacterial culture reached an OD_600_ of 0.075 (approximately 3 h), early-exponential OMVs were collected after the culture reached an OD_600_ of 0.10–0.12 (approximately 6 h), and late-exponential OMVs were collected after the culture reached an OD_600_ of 0.75–1.00 (approximately 20 h). The cultures were grown to the specified OD_600_. The first of these cultures was centrifuged at 10,000× *g* for 10 min at 4 °C to pellet the cells. The vesicles produced up to that point were removed from the supernatant using a 0.2 µm filter, followed by 2 h of centrifugation at 35,000× *g* and 4 °C. The resulting OMV-free supernatant was used to resuspend a bacterial pellet (grown in parallel to the same point, centrifuged at 3800× *g* for 10 min at room temperature), and this culture was grown until the next time point was reached. The process was continued through the late exponential phase.

### 4.4. Lipid Concentration Determination

The lipid content of the OMV preparations was measured using the FM^TM^ 4–64 dye (ThermoFisher Scientific, Waltham, MA, USA). First, 50 µL of each sample was incubated with FM^TM^ 4–64 (0.1 mg/mL) for 15 sec. Following incubation, the fluorescence was measured on a Tecan plate reader at an excitation wavelength of 515 nm and an emission wavelength of 640 nm.

### 4.5. THP-1 Cell Viability

To measure the OMV-mediated cytotoxicity, OMVs were added at equal lipid concentrations to a THP-1 cell culture. The cellular metabolic activity was then measured using a 3-(4,5-dimethylthiazol-2-yl)-2,5-diphenyltetrazolium bromide (MTT)-based assay. To each sample well, 0.48 mg/mL of thiazolyl blue tetrazolium bromide (Sigma-Aldrich) in PBS was added and incubated for 4 h at 37 °C. The media was removed, and the precipitate was dissolved in dimethyl sulfoxide (DMSO); the absorbance was then measured at a wavelength of 570 nm. The absorbance from treated cells compared to the absorbance from non-treated cells was used as a measure of cell viability.

### 4.6. Immunoblot

To determine the LtxA composition of *A. actinomycetemcomitans* OMVs, an immunoblot was performed by first lysing samples with 0.5% Triton-X 100 (Sigma-Aldrich). Next, 2 µL volumes of the samples, each containing 0.2 mg/mL of total protein, determined by the absorbance at a wavelength of 280 nm (A_280_), were blotted onto a nitrocellulose membrane (Bio-Rad). Known concentrations of purified LtxA were also blotted onto the membrane as standards. The membrane was dried and blocked in blotto solution (5% dried milk in Tris-buffered saline with 0.1% Tween-20 (TBST)) for 1 h. The presence of LtxA was detected with a monoclonal anti-LtxA antibody [38], followed by horseradish peroxidase-conjugated goat anti-mouse (GAM-HRP) for 1 h. The blot was imaged using a SuperSignal™ West Dura substrate (ThermoFisher).

### 4.7. Dynamic Light Scattering (DLS)

An ALV/CGS-3 goniometer system was used to determine the diameter distributions of the OMVs. Samples were suspended in PBS and measured for three minutes at a wavelength of 632.8 nm and a scattering angle of 90°. Size distributions were calculated using ALV software (ALV-5000/E, ALV-GmbH, Langen, Germany, 2001), using a number-weighted regularized fit with the coated sphere assumption (membrane thickness r* = 5 nm) [54]. The number-weighted fit corrects for the fact that larger particles scatter more light than smaller particles by normalizing the particles by count rather than scattering.

### 4.8. Size Exclusion Chromatography

SEC was used to separate OMVs by size; a 1.5 cm × 50 cm column (bed volume 85 mL) was packed with Sephacryl™ S-1000 superfine resin (GE Healthcare, Chicago, IL, USA) [55] and equilibrated with two bed volumes of PBS. A 2 mL OMV sample was loaded, eluted with PBS, and 1 mL fractions were collected. Fractions were analyzed for lipid, LtxA, and surface-associated DNA concentrations, as described below.

### 4.9. Surface-Associated DNA Content

The amount of DNA on the surface of the OMV fractions was measured using TOTO™-1 Iodide (ThermoFisher Scientific). A TOTO™-1 stock solution was made by mixing 1 µL of TOTO™-1 (1 mM in DMSO) with 250 µL of Tris-EDTA buffer (TE buffer, 0.1 M of Tris, 0.1 M of EDTA, pH 8.0). The stock solution was mixed in equal volume ratios with the OMV fractions and incubated for 10 min at room temperature. Following incubation, the fluorescence was measured using a Quantamaster^®^ 400 spectrofluorometer (PTI Horiba, Edison, NJ) with an excitation wavelength of 514 nm and an emission wavelength of 533 nm.

### 4.10. Protein Characterization

The LtxA concentration in each SEC fraction was measured using an enzyme-linked immunosorbent assay (ELISA). Fractions were incubated in a MaxiSorp Immuno 96-well plate (ThermoFisher Scientific) for 3 h, washed five times with ELISA wash buffer (25 M of Tris, 150 mM of sodium chloride, 0.1% fatty-acid-free bovine serum albumin (BSA), and then blocked in 1% BSA in the same buffer. The plate was then incubated with an anti-LtxA antibody [38] in 1% BSA/buffer overnight at 4 °C. Following five washes with ELISA wash buffer, the plate was incubated in goat anti-mouse horseradish peroxidase (GAM-HRP) at a 1:5000 ratio (SouthernBiotech, Birmingham, AL, USA). Lastly, the plate was imaged using a 1-Step^TM^ Ultra TMB ELISA substrate solution (ThermoFisher Scientific) until a signal appeared, and then the reaction was stopped using 2 M of sulfuric acid. The absorbance at 450 nm was measured on a Tecan plate reader. The resulting absorbance of each fraction was divided by the summed absorbances to calculate the percentage of total LtxA in each fraction.

LtxA content was also analyzed by Western blotting. Sodium dodecyl sulfate polyacrylamide gel electrophoresis (SDS-PAGE) was performed using 7.5% acrylamide gels. Western blotting for LtxA was accomplished by transferring the proteins to a nitrocellulose membrane overnight. The blots were washed three times in Tris-buffered saline with 0.1% tween (TBST) and then blocked with a blotto solution (5% dried milk in TBST) for 1 h. LtxA was then detected using a monoclonal anti-LtxA antibody [38] overnight at 4 °C, followed by GAM-HRP for 1 h. The blot was imaged using a SuperSignal™ West Dura substrate (ThermoFisher). To measure the amount of outer membrane protein-A (OmpA) in each fraction, a similar procedure was used, with the anti-Gram-negative OmpA antibody (111228, Antibody Research Corporation, St. Charles, MO, USA) at a final concentration of 0.24 μg/mL, followed by goat anti-rabbit horseradish peroxidase (GAR-HRP, SouthernBiotech). Densitometry analysis on the blots was accomplished using ImageJ [56].

### 4.11. LtxA Binding Experiments

To determine the role of LPS in LtxA’s association with OMVs, the OMVs were treated with PMB at a concentration of 4 mg/mL at 37 °C for 1 h [23] before separation by SEC, as described above. To determine the role of DNA in LtxA’s association with OMVs, the OMVs were treated with DNase I (Sigma-Aldrich) at a final concentration of 100 U/mL at 37 °C for 1 h [22]. Following treatment, OMVs were separated by SEC, as described above.

### 4.12. Statistical Analysis

Statistical analysis was performed with Sigma-Plot using an unpaired two-tailed student’s *t*-test, where *p* values ≤ 0.01 were considered statistically significant.

## Data Availability

Data are available upon request.

## Supplementary Materials

The following supporting information can be downloaded at https://www.mdpi.com/article/10.3390/toxins16030138/s1. Figure S1: Representative growth curve of JP2 bacteria and JP2 bacteria in depleted media; Figure S2. LtxA content in the supernatant of a JP2 culture.
