# Supplementary material for: Heterogeneity of Size and Toxin Distribution in Aggregatibacter actinomycetemcomitans Outer Membrane Vesicles
Source: Toxins (Basel). 2024 Mar 7;16(3):138. doi: 10.3390/toxins16030138 (PMC10974469; doi:10.3390/toxins16030138)
Supplement: Supplementary file 1 [file toxins-16-00138-s001.zip › toxins-2864818-supplementary.pdf]

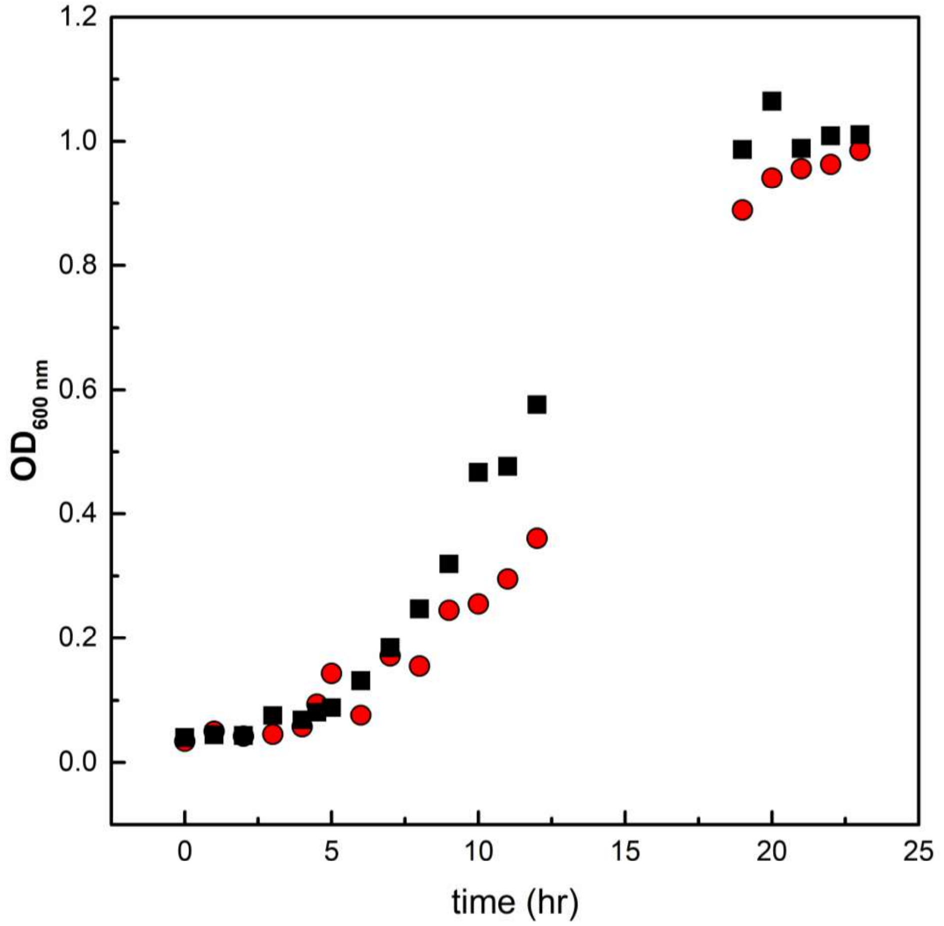

Figure S1. Representative growth curve of JP2 bacteria and JP2 bacteria in depleted media. JP2 bacteria were grown normally (black squares). After 6 hr, JP2 bacteria were pelleted and resuspended in depleted, OMV free media (red circles)

Std 2 hr 3 hr 4 hr 5 hr 6 hr 8 hr 9 hr 10 hr 12 hr

250 kDa
150 kDa
100 kDa
75 kDa
37 kDa

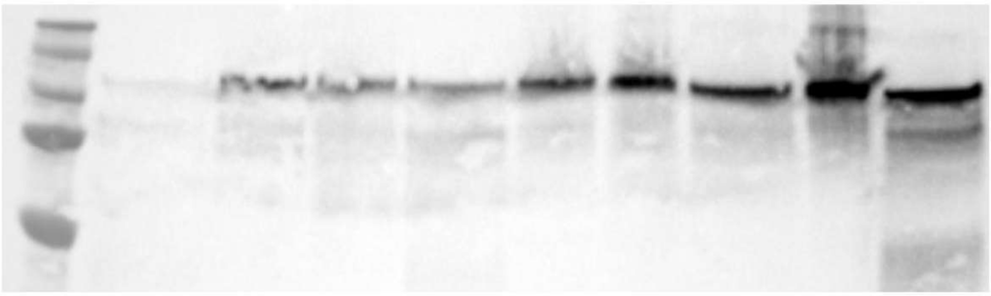

Figure S2. LtxA content in the supernatant of a JP2 culture. An aliquot of the JP2 supernatant was removed at the time points noted. The proteins were precipitated using cold ethanol, and the samples were analyzed by Western blot using an anti-LtxA antibody.
